# Evolution of *TOP1* and *TOP1MT* Topoisomerases in Chordata

**DOI:** 10.1007/s00239-022-10091-z

**Published:** 2023-01-18

**Authors:** Filipa Moreira, Miguel Arenas, Arnaldo Videira, Filipe Pereira

**Affiliations:** 1 Interdisciplinary Centre of Marine and Environmental Research (CIIMAR), University of Porto, Terminal de Cruzeiros do Porto de Leixões, Av. General Norton de Matos S/N 4450-208, Matosinhos, Portugal; 2 ICBAS - Instituto de Ciências Biomédicas de Abel Salazar, Universidade do Porto, Rua Jorge de Viterbo Ferreira 228, 4050-313 Porto, Portugal; 3 Department of Biochemistry, Genetics and Immunology, University of Vigo, 36310 Vigo, Spain; 4 CINBIO, Universidade de Vigo, 36310 Vigo, Spain; 5 Galicia Sur Health Research Institute (IIS Galicia Sur), 36310 Vigo, Spain; 6 IBMC-Instituto de Biologia Molecular e Celular, Universidade do Porto, Porto, Portugal; 7 i3S-Instituto de Investigação e Inovação em Saúde, Universidade do Porto, Porto, Portugal; 8 IDENTIFICA Genetic Testing, Rua Simão Bolívar 259 3º Dir Tras, 4470-214 Maia, Portugal; 9 Centre for Functional Ecology, Department of Life Sciences, University of Coimbra, Calçada Martim de Freitas, 3000-456 Coimbra, Portugal

**Keywords:** Type IB topoisomerases, Molecular phylogeny, Purifying selection, Neanderthals, Functional divergence

## Abstract

Type IB topoisomerases relax the torsional stress associated with DNA metabolism in the nucleus and mitochondria and constitute important molecular targets of anticancer drugs. Vertebrates stand out among eukaryotes by having two Type IB topoisomerases acting specifically in the nucleus (TOP1) and mitochondria (TOP1MT). Despite their major importance, the origin and evolution of these paralogues remain unknown. Here, we examine the molecular evolutionary processes acting on both TOP1 and TOP1MT in Chordata, taking advantage of the increasing number of available genome sequences. We found that both TOP1 and TOP1MT evolved under strong purifying selection, as expected considering their essential biological functions. Critical active sites, including those associated with resistance to anticancer agents, were found particularly conserved. However, TOP1MT presented a higher rate of molecular evolution than TOP1, possibly related with its specialized activity on the mitochondrial genome and a less critical role in cells. We could place the duplication event that originated the *TOP1* and *TOP1MT* paralogues early in the radiation of vertebrates, most likely associated with the first round of vertebrate tetraploidization (1R). Moreover, our data suggest that cyclostomes present a specialized mitochondrial Type IB topoisomerase. Interestingly, we identified two missense mutations replacing amino acids in the Linker region of TOP1MT in Neanderthals, which appears as a rare event when comparing the genome of both species. In conclusion, TOP1 and TOP1MT differ in their rates of evolution, and their evolutionary histories allowed us to better understand the evolution of chordates.

**Supplementary Information:**

The online version contains supplementary material available at 10.1007/s00239-022-10091-z.

## Introduction

DNA topoisomerases introduce reversible breaks in the DNA phosphodiester backbone allowing for modifications in DNA topology during DNA replication, recombination, transcription and chromosome condensation (Pommier et al. 2022, 2016). Concerning Type I topoisomerases, they are monomeric and cleave one DNA strand at a time without requiring an energy cofactor. These topoisomerases are traditionally classified into two groups (Type IA and Type IB) without sequence or structural similarity. Indeed, while Type IA breaks the DNA by forming a covalent bond to the 5′ end, Type IB binds covalently to the 3′ end of the break (Capranico et al. 2017; Cheng et al. 1998; Redinbo et al. 1998).

Type IB topoisomerases were found in some bacteria and Poxviruses and in eukaryotes (Champoux 2001; Forterre et al. 2007). All eukaryotes have at least one topoisomerase I (TOP1) for relaxing both negative and positive supercoils in front of moving polymerases during replication and transcription. Studies in yeast suggest that a single TOP1 may act in both the nuclear and mitochondrial genomes (de la Loza and Wellinger 2009; Wang et al. 1995). However, a second Type IB topoisomerase (TOP1MT) was identified in vertebrates, encoded in the nuclear genome. The TOP1MT exclusively localizes to mitochondria via a mitochondrial targeting sequence (MTS) at its N-terminal domain (Zhang et al. 2001). Among model organisms, TOP1 is essential for mouse and fruit fly development (Lee et al. 1993; Morham et al. 1996). TOP1MT seems to be dispensable for mouse development, but its absence causes increased negative supercoiling of mitochondrial DNA (mtDNA) and affects cellular energy metabolism (Douarre et al. 2012; Zhang et al. 2014) by interfering with biological processes such as liver regeneration (Khiati et al. 2015). Despite the biological relevance of both genes, their origin and molecular evolutionary patterns are still unknown.

**Fig. 1:**
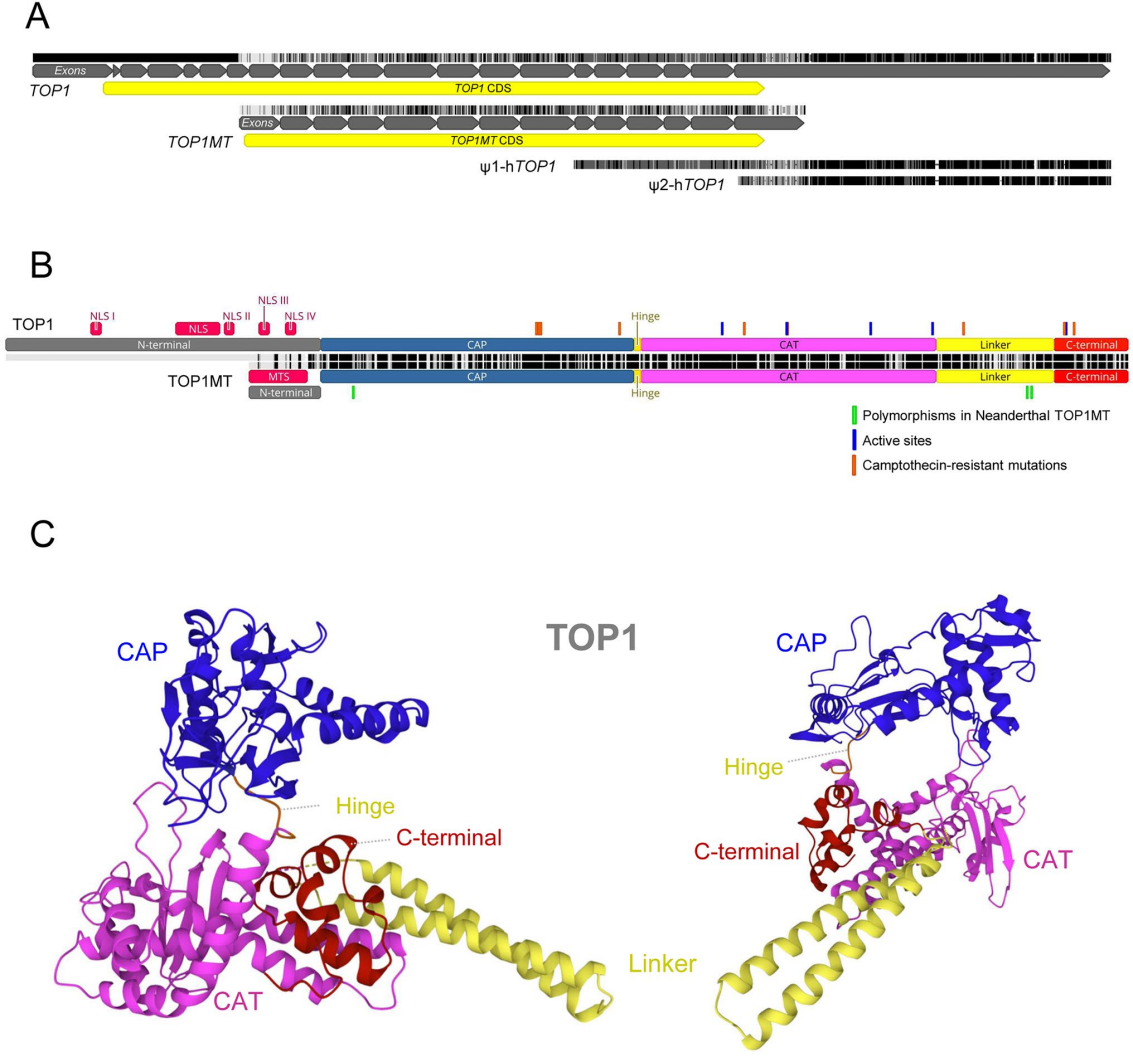
Organization of human nuclear (TOP1) and mitochondrial (TOP1MT) DNA topoisomerases I. **A** Multiple sequence alignment of human *TOP1* and *TOP1MT* mRNA sequences and the two *TOP1* pseudogenes identified in chromosomes 1 (ψ1-h*TOP1*) and 22 (ψ2-h*TOP1*). **B** Pairwise alignment of TOP1 and TOP1MT protein sequences, annotated with the most relevant protein domains and sites. **C** Illustrative representation of the human TOP1 protein structure with major domains highlighted

In humans, the *TOP1* gene is located in the chromosome region 20q12 (Juan et al. 1988) and encodes a 91 kDa protein with 765 amino acids. Two *TOP1* pseudogenes have been identified on chromosomes 1 (ψ1-h*TOP1*) and 22 (ψ2-h*TOP1*) resulting from truncated mRNA transcripts of the active gene (Fig. 1A) (Yang et al. 1990). The *TOP1MT* gene maps to chromosome region 8q24 resulting in a 70 kDa protein with 601 amino acids (Zhang et al. 2001). Although *TOP1* has 21 exons and *TOP1MT* has 14 exons, the terminal 13 exons are conserved between both genes (Zhang et al. 2004).

Considering the molecular structure and sequence conservation, TOP1 and TOP1MT proteins are organized into four distinct domains: N-terminal, Core, Linker and C-terminal domains (Fig. 1B, C). The N-terminal domain is poorly conserved across species and varies considerably when comparing both proteins. In particular, the TOP1 N-terminal is highly charged and relatively unstructured, being dispensable for the enzyme activity, mediates protein–protein interactions and includes nuclear localization signals (NLSs) (Alsner et al. 1992; Mo et al. 2000; Palle et al. 2008). The TOP1MT N-terminal is much shorter than that from TOP1 and includes a MTS. The core domain is highly conserved and contains essential catalytic residues, being connected to the C-terminal domain by a poorly conserved Linker region formed by an extended pair of α-helices. TOP1 forms a toroidal fold with two modules entrapping the DNA molecule, a capping module matching the first half of the core domain (CAP domain or core sub-domains I and II) and a catalytic module comprising the second half of the core domain (CAT domain or core sub-domain III), the Linker and the C-terminal domain (Redinbo et al. 1998; Stewart et al. 1998; Takahashi et al. 2022). The catalytic module includes several active sites relevant for the protein activity (Champoux 2001). The Hinge is a five-residue loop connecting the capping and catalytic modules whose flexibility permits the opening/closing of the enzyme and the entry of DNA (Takahashi et al. 2022). The C-terminal domain is highly conserved and includes the Tyr723 active site which forms a transient phosphotyrosyl linkage to one DNA strand, catalysing changes in DNA topology (Stewart et al. 1996).

Importantly, TOP1 is the target of the camptothecin family of anticancer agents that binds to and reversibly stabilizes the covalent TOP1-DNA complex, resulting in double stranded DNA breaks and apoptosis, preferentially in cancer cells that often overexpress TOP1 (Pommier 2006; Pommier et al. 2010). TOP1MT is also sensitive to camptothecin agents, but it is not an in vivo target due to the alkaline mitochondria matrix that inactivates the drug (Tua et al. 1997; Zhang et al. 2001; Zhang and Pommier 2008). However, several mutations in TOP1 are known to impact the efficacy of camptothecin (Chrencik et al. 2004; Cretaio et al. 2007; Saleem et al. 2000).

Previous works have compared Type IB topoisomerases from different species, but often focused on a specific section of the protein or explored only a few animal species [e.g., (Champoux 1998; Takahashi et al. 2022; Zhang et al. 2004)]. Here, we present a detailed examination of the evolutionary history of Type IB topoisomerases using a variety of animals that represent the main taxonomic groups of Chordata. In particular, we evaluated the molecular evolution and adaptation processes and the origin of the TOP1 and TOP1MT paralogues in vertebrates.

## Material and Methods

### TOPIB Sequences

TOPIB protein sequences from the main Metazoa phyla were retrieved from the NCBI non-redundant protein sequences (nr) database via the protein–protein BLAST (blastp) suite, using as query sequences from species close to the target taxonomic group (Supplementary Fig. S1). Short sequences with less than half of the average of TOPIB length were ignored since they often represent partial protein sequences derived from gaps in assembled genomes in which the contigs do not cover the complete genomic region. Possibly by the same reason, we fail to detect one or both the paralogues in the sequenced genome of some species.

Denisovan and Neanderthal *TOP1* and *TOP1MT* sequences were downloaded from the UCSC Genome Browser (http://genome.ucsc.edu/) (Kent et al. 2002). All BAM reads for tracks *Denisova* and *Neanderthal Cntgs* matching the Human Mar. 2006 (NCBI36/hg18) chr20:39,090,876–39,186,540 (*TOP1*) and chr8:144,462,903–144,488,425 (*TOP1MT*) were downloaded. The BAM reads from each track were then reassembled against the human *TOP1* (NC_000020.11) and *TOP1MT* (NC_000008.11) reference sequences using Geneious v2022.1.1 (http://www.geneious.com). We only considered a variable position in Denisovan and Neanderthal genomes when: (1) at least two reads overlap in that position; (2) the variant represents more than 75% of all the reads and (3) the difference is not at the end of a read. The variations between modern humans and Neanderthals were also confirmed in the assembly available at The Neandertal Genome Project (http://neandertal.ensemblgenomes.org).

### TOPIB Sequence Alignments

The TOPIB protein sequences were aligned with the Geneious alignment in three datasets: Metazoa (*n* = 161), Chordata TOP1 (*n* = 48) and Chordata TOP1MT (*n* = 48). The conservation across the alignments was measured with the percentage of pairwise identity (PI) that compares base pairs at every site. The same species were used in the Chordata alignments to avoid biases and facilitate the comparison of results. The coding domain sequences (CDS) of the orthologues of human TOP1 (ENSG00000198900) and TOP1MT (ENSG00000184428) were obtained from the Ensembl Genome Server (Hunt et al. 2018).

### Phylogenetic Analyses

We analysed the TOPIB duplication events in chordates with a phylogenetic tree built with 37 protein sequences from Cephalochordata, Tunicata and Vertebrata species, and considering *Acanthaster planci* and *Strongylocentrotus purpuratus* (Echinodermata) as outgroups. We used Gblocks 0.91b server, running on Phylogeny.fr (Dereeper et al. 2008), to remove poorly aligned positions under the settings for a less stringent selection (Castresana 2000; Talavera and Castresana 2007). The best-fitting amino acid substitution model of evolution (LG + I + G4 + F) was determined with ModelTest-NG (Darriba et al. 2020; Flouri et al. 2015). Next, we build a Bayesian phylogenetic tree with MrBayes v3.2.7a (Huelsenbeck and Ronquist 2001; Ronquist and Huelsenbeck 2003) running on the CIPRES Science Gateway v3.3 (Miller et al. 2010). The Metropolis-coupled Markov chain Monte Carlo (MCMC) process was set with two independent runs, each with four independent chains that ran simultaneously during 4,000,000 iterations. The average standard deviation of split frequencies of the final tree was 0.002339, indicating convergence among the independent runs. A burn-in value of 0.25 was applied following the program recommendation. The resulting phylogenetic tree was edited with FigTree v1.4.3 (http://tree.bio.ed.ac.uk/software/figtree).

### Evaluation of Selection

Molecular adaptation signatures in TOP1 and TOP1MT protein-coding sequence alignments were evaluated with the nonsynonymous/synonymous substitution rates ratio (*dN*/*dS*) (Del Amparo et al. 2021; Jeffares et al. 2015). First, we selected the best-fitting substitution model of DNA evolution and reconstructed a maximum likelihood (ML) phylogenetic tree. Next, we estimated *dN*/*dS* under a ML method, considering the reconstructed phylogenetic tree, implemented in the evolutionary framework Hyphy (Kosakovsky Pond and Frost 2005; Kosakovsky Pond et al. 2020). In particular, we applied the single-likelihood ancestor counting (SLAC) method for the *dN*/*dS* estimation, which has an accuracy similar to that from other likelihood-based methods and includes statistical evaluations (Kosakovsky Pond and Frost 2005).

### Template of TOPIB Protein Structure

We considered as an illustrative template of the human TOPIB protein structure, the protein structure of the Protein Data Bank (PDB) (Berman et al. 2000) with code 1A36 (Stewart et al. 1998). The structure was analysed with Mol* (Sehnal et al. 2021) and RCSB PDB.

## Results and Discussion

### TOP1 and TOP1MT Paralogues Originated in the First Round of Vertebrate Tetraploidization (1R)

**Fig. 2:**
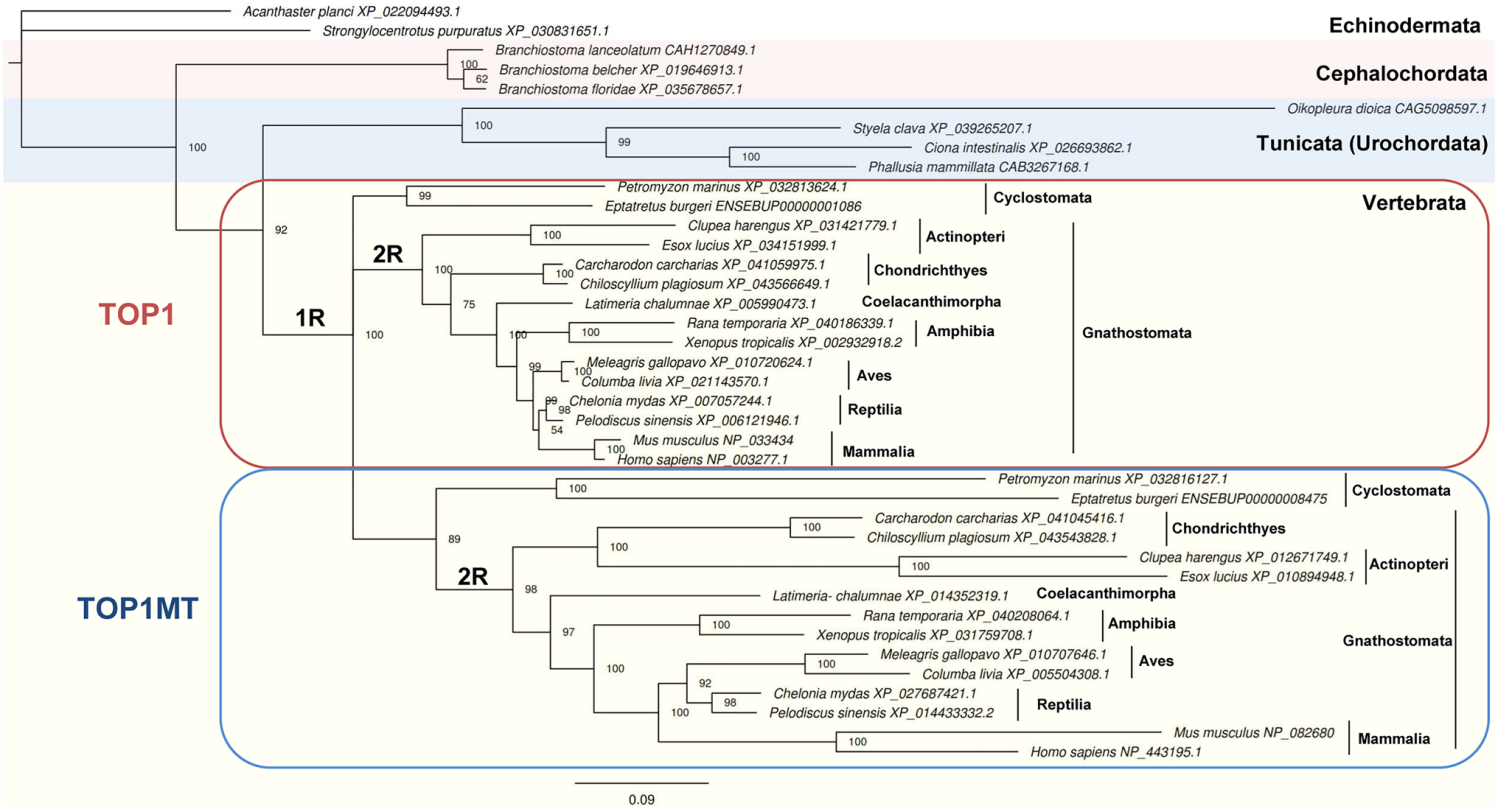
Phylogenetic analysis of TOPIB in chordates. Bayesian phylogenetic tree built with an alignment of 37 TOPIB protein sequences from chordates and considering two Echinodermata species as outgroup. The Bayesian posterior probabilities are shown on the internal nodes. The scale bar indicates substitutions per site. The putative occurrence of two rounds of tetraploidization (1R and 2R) is indicated

Previous works have shown that TOPIB topoisomerases are ubiquitous in eukaryotes, and that only vertebrates have two *TOPIB* paralogues, named *TOP1* and *TOP1MT* (Forterre et al. 2007; Zhang et al. 2004). Our extensive search for *TOPIB* genes in the genome of all available chordates only retrieved paralogues in the cyclostomes (jawless vertebrates) and gnathostomes (jawed vertebrates), confirming the previous claiming that *TOPIB* paralogues only occur in vertebrates (Zhang et al. 2004). Our phylogeny placed cephalochordates at the root of Chordata (Fig. 2). The Tunicata (Urochordata) and Vertebrata form a clade known as Olfactores (Delsuc et al. 2006; Putnam et al. 2008; Satoh et al. 2014). The fast-evolving *Oikopleura dioica* forms a particularly long branch, as we previously found for TOPIIA (Moreira et al. 2022)*.*

The timing of the duplication event that gave rise to both paralogues remains unclear, particularly considering that the origin of vertebrates is associated with several gene and genome duplication events. Two rounds of tetraploidization, known as 1R and 2R, are believed to have occurred early in vertebrate evolution (Ohno 2013; Smith and Keinath 2015; Van de Peer et al. 2009). The timing of the tetraploidization events is still a matter of debate, but it was recently proposed that 1R preceded the divergence between cyclostomes and gnathostomes and 2R only occurred in gnathostomes (Aase-Remedios and Ferrier 2021; Nakatani et al. 2021; Simakov et al. 2020). Previous works observed that vertebrata *TOP1* and *TOP1MT* form two separate clusters (Forterre et al. 2007; Wang et al. 2009; Zhang et al. 2007), but were performed without sequences from cyclostomes. Our search for *TOPIB* genes in cyclostomes allowed us to retrieve two complete *TOPIB* sequences in two species, *Petromyzon marinus* and *Eptatretus burgeri.* We also noticed the presence of at least two paralogues in other cyclostomes (e.g., *Lethenteron camtschaticum*, *Entosphenus tridentatus*), but the genomic sequences were incomplete and thus were not used in the phylogenies. Therefore, it is likely that cyclostomes have at least two TOPIB paralogues, as observed in other vertebrates. In this concern, the *TOPIB* paralogues from *P. marinus* and *E. burgeri* did not cluster together in our phylogeny (Fig. 2). Instead, one pair clusters with TOP1MT sequences. Indeed, these two paralogues also display long branches, which are typical for the fast-evolving TOP1MT. Therefore, our analyses suggest that cyclostomes have a mitochondrial Type IB topoisomerase. The other pair of TOPIB paralogues from *P. marinus* and *E. burgeri* split from Gnathostomata TOP1 and TOP1MT at similar times. Our analysis is compatible with the idea that the duplication event that originated *TOP1* and *TOP1MT* is related with the first round of tetraploidization (1R). In this situation, *TOP1* and *TOP1MT* originated during the whole genome duplication in the early vertebrate evolution. The paralogues then diverged independently during the evolution of vertebrates, clustering in two separate branches (Fig. 2). The main difference between the phylogeny of the two genes is the placement of TOP1 from cyclostomes, which does not cluster with TOP1 from gnathostomes, as in the TOP1MT clade. Further analyses with additional sequences from Cyclostomata are necessary to better define the evolutionary history of these genes.

The specialization for acting on mtDNA may have occurred early in the radiation of vertebrates. In this concern, we previously identified that TOPIIA paralogues (*TOP2A* and *TOP2B*) present a different origin within chordates (Moreira et al. 2022). Here, we found that *TOP2A* and *TOP2B* paralogues from Cyclostomata cluster together in a separate branch from all Gnathostomata paralogues. Altogether, our findings suggest that the different classes of topoisomerases present different evolutionary histories in chordates.

### Strong Purifying Selection Acting on TOP1 and TOP1MT

We estimated the *dN*/*dS* ratio to evaluate selection acting on TOP1 and TOP1MT paralogues of chordates (Table 1). We found that both genes present genetic signatures of negative (purifying) selection (*dN*/*dS* < 1), as noticed before in other topoisomerases (TOP3B, TOP2A, TOP2B) (Moreira et al. 2021, 2022). The paralogue pairs TOP1/TOP1MT and TOP2B (*dN*/*dS* = 0.156) / TOP2A (*dN*/*dS* = 0.238) (Moreira et al. 2022) presented higher *dN*/*dS* ratios than TOP3B (*dN*/*dS* = 0.076) (Moreira et al. 2022), which has no paralogue.

**Table 1:** Selection pressure in *TOP1* and *TOP1MT*

Gene	Dataset	*n*	Best substitution model	Global dN/dS*	Pairwise identity (%)
*TOP1*	Chordata	74	SYM + G	0.154 [0.147–0.162]	82.0
*TOP1MT*	Chordata	74	SYM + I + G	0.307 [0.300–0.315]	61.3

*Global (entire sequences) *dN*/*dS* including the 95% confidence interval. Positively selected sites (PSS) were not detected

Paralogues can exhibit asymmetric rates of sequence evolution (Conant and Wagner 2003; Scannell and Wolfe 2008; Van de Peer et al. 2001). The strength of negative selection was higher in TOP1 (*dN*/*dS* = 0.154) than in TOP1MT (*dN*/*dS* = 0.307). Indeed, TOP1 also exhibits a lower diversity compared with TOP1MT (Table 1). The essential activity of TOP1 across species (Lee et al. 1993; Morham et al. 1996) in different biological processes can explain its relatively high conservation. On the other hand, TOP1MT presents the highest *dN*/*dS* ratio among all the topoisomerases studied by us (Moreira et al. 2021, 2022). Although it still evolved under negative selection, TOP1MT seems more permissive to accept amino acid changes than other topoisomerases. The higher diversity estimated in TOP1MT (in comparison to TOP1) can also be observed in the Chordata phylogeny, where TOP1MT branches are considerably longer than those for TOP1 (Fig. 2). The fast rate of change in *TOP1MT* can explain why finding orthologues for this gene is difficult. For example, the Ensembl genome browser only recognizes 77 orthologues for *TOP1MT*, in comparison with the 272 orthologues identified for TOP1 (accessed in April 2022). TOP1MT was also recognized as the only topoisomerase with highly frequent single nucleotide variants (SNVs) in the human population (Zhang et al. 2017). It was speculated that TOP1MT varies more than other topoisomerases due to several factors: (*i*) it is a nonessential gene under less constraints to mutate; (*ii*) it is in a subtelomeric end of a chromosome and/or (*iii*) it is a relatively recent gene under adaptation to its activity in mitochondria (Zhang et al. 2017). Thus, the observed pattern can be the result from a combination of those factors. Comparing with our previous results, *TOP2B* and *TOP2A* are more conserved than *TOP1MT* despite being also paralogues that originated early in vertebrate evolution (Moreira et al. 2022). Thus, we believe that these paralogues could be a good comparative model to study *TOP1MT* in future investigations.

### Two Missense Mutations Identified in the Neandertals TOP1MT Linker Region

**Fig. 3:**
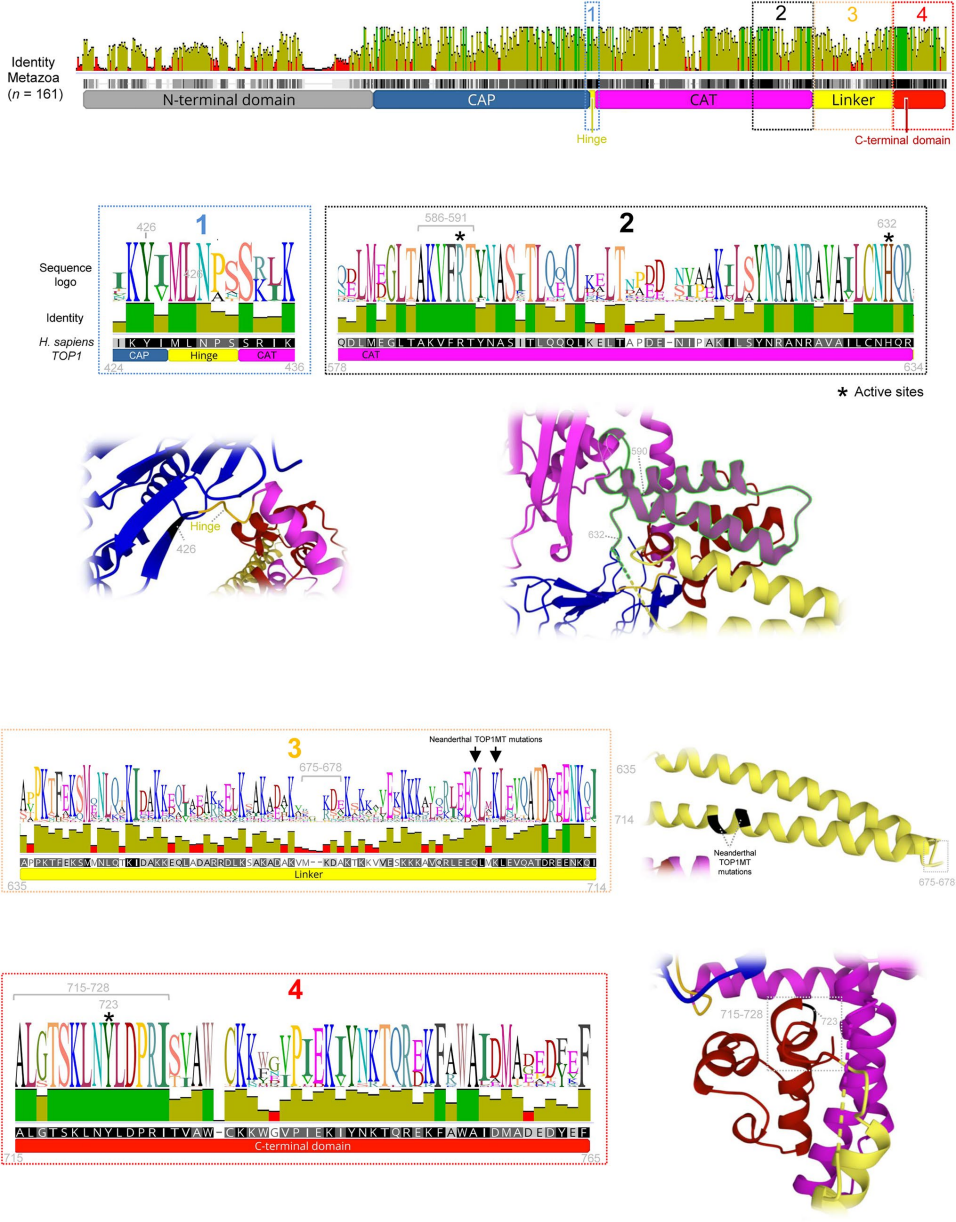
Structural conservation of TOPIB. The sequence identity plot was estimated from the 161 TOPIB protein sequences of the major chordate groups. The most conserved positions are indicated with brown bars, while the less conserved positions are shown using red bars. The sequence logo and an illustration of the protein structure for the highlighted regions are included (Color figure online)

Neanderthals and Denisovans are extinct groups of hominins that inhabited Eurasia until around 40,000 years ago (Green et al. 2010; Reich et al. 2010). Previous works identified a few amino acid changes among modern humans and other hominins, some of which may have contributed to unique human traits (Green et al. 2010; Kuhlwilm and Boeckx 2019). Here, we searched for sequence differences in coding regions among modern human, Denisovan and Neanderthal *TOP1* and *TOP1MT* genes. However, we did not identify polymorphic positions in *TOP1* coding regions covered by Neanderthals or Denisovans sequence reads. On the contrary, we identified three nucleotide differences in the coding regions of *TOP1MT* (Table 2). A silent mutation in the CAP TOP1MT domain occurred in the human lineage. Next, two missense mutations were identified in the Neanderthal lineage. In particular, the mutations involved changes in two close amino acid positions (533 and 536) that belong to the Linker region (Fig. 3). Notice that the occurrence of missense mutations between modern humans and Neanderthals is rare (Green et al. 2010; Kuhlwilm and Boeckx 2019). When comparing present-day human and Neanderthals, Kuhlwilm and Boeckx (2019) identified 647 amino acid-changes in 571 genes. Among those genes, only 68 had two or more amino acid changes. Assuming that humans have 19,969 genes (Nurk et al. 2022), only 0.34% of those genes have more than one amino acid change, making it a rare event.

**Table 2:** Sequence variants identified in *TOP1MT* coding sequences among modern humans (*H. sapiens*), Denisovan (*Denis*) and *Homo neanderthalensis* (*Neand*)

Gene	*Homo sapiens* reference sequences						Ancestral State*		Variant in Denisovan and Neanderthal		Mutational event				Pairwise Identity (%) in Chordata
	Sequence	Genome position	nt	Protein position	aa	Protein domain	nt	aa	Species	nt	Probable event	Lineage	Type	Amino acid replacement	
*TOP1MT*	NC_000008.11	143,331,246	G	72	Asp	CAP	A	Asp	Denis	A	A > G	*H. sapiens*	Silent	–	26.4
		143,310,173	T	533	Gln	Linker	T	Gln	Neand	C	T > C	Neand	Missense	Gln-Arg	92.7
		143,310,165	G	536	Gln	Linker	G	Gln	Neand	T	G > T	Neand	Missense	Gln-Lys	84.7

*Nucleotide in *Pan paniscus* and *Gorilla gorilla*

Two mutations occurring in the same sequence read seems particularly improbable. However, we identified the mutations in several reads, including both our assembly and the assembly available at the Neandertal Genome Project (Supplementary Fig. S2). Moreover, we fail to align the Neandertal reads with any other available sequence in GenBank, including *TOP1* gene and pseudogenes, which excludes a possible misplacement of reads from those regions in *TOP1MT*. The two mutations involved amino acids with different physicochemical properties. In particular, two glutamines (polar uncharged side chain) were replaced by an arginine and a lysine (positively charged, basic, side chain). These different properties could affect the protein function, but further experimental analyses are required to corroborate this possibility. We previously identified two missense mutations in TOP2A when comparing present-day humans and Neanderthals (Moreira et al. 2022). It is interesting to note that missense mutations were only identified in the two topoisomerases (TOP1MT and TOP2A) that are less conserved in chordates, which supports the credibility of the identified sequence differences. The sequencing of additional Neanderthal and Denisovan samples will allow us to confirm if these sequence variations were fixed among these species.

### Relevant TOP1 and TOP1MT Sites for Catalytic Activities Tend to be Conserved Across Animals

The alignment of TOP1 and TOP1MT protein sequences from 48 representative chordate species confirms that TOP1MT (77% of pairwise sequence identity) is less conserved than TOP1 (sequence identity of 83.6%) (Fig. 3, Table 3). This result agrees with the long branches of TOP1MT in the Chordata phylogeny (Fig. 2) and its higher genetic diversity (Table 2). The N-terminal domain is the less conserved region in both proteins (sequence identities of 64.1% in TOP1 and 53.3% in TOP1MT), as noticed since the first studies on TOP1 (Champoux 1998, 2001; Stewart et al. 1996). The function of the N-terminal domain remains poorly understood, partially due to a lack of structural information. However, it is dispensable for the catalytic activity of the enzyme (Alsner et al. 1992), suggesting that it could accept mutations without compromising the protein activity. Moreover, the N-terminal domain mediates TOP1 interactions with other proteins (Czubaty et al. 2005). These protein–protein interactions might experience different co-evolution processes among species that could explain the poor sequence conservation of the domain. The protein–protein binding regions identified in N-terminal domains of TOP1 (NLSs) and TOP1MT (MTS) are also poorly conserved, possibly due to evolution driven by different species requirements. Only TOP1 NLS-II and NLS-IV are relatively conserved in chordates (Table 3).

**Table 3:** Organization and conservation of TOP1 and TOP1MT protein domains and relevant sites. The percentage of pairwise identity was obtained in an alignment with all metazoans and with chordates alone

Protein domains		TOP1			TOP1MT			TOPIB
		Human reference		Chordata (*n* = 48)	Human reference		Chordata (*n* = 48)	Metazoa (*n* = 161)
		Position	Length (aa)	Pairwise identity (%)	Position	Length (aa)	Pairwise identity (%)	Pairwise identity (%)
Complete protein		1–765	765	83.6	1–601	601	77	70.5
N-terminal domain		1–213	213	64.1	1–49	49	53.3	41.9
Core domain	CAP	214–427	214	89	50–263	214	77.3	69.8
	Hinge	428–432	5	98.3	264–268	5	84.1	84.8
	CAT	433–635	202	94.5	269–470	202	81.4	78.4
Linker		636–714	80	87	471–550	80	63.1	57.3
C-terminal domain		715–765	51	93	551–601	51	85.4	82.5
N-terminal localization signals	TOP1 NLS	117–146	30	56.8	–	–	–	–
	TOP1 NLS-I	59–65	7	62.9	–	–	–	–
	TOP1 NLS-II	150–156	7	86.9	–	–	–	–
	TOP1 NLS-III	174–180	7	35.4	–	–	–	–
	TOP1 NLS-IV	192–198	7	79.2	–	–	–	–
	TOP1MT MTS	–	–	–	1–40	40	34.8	–
Active sites	CAT	488	R	100	324	R	91.8	97.5
	CAT	532	K	100	368	K	95.8	100
	CAT	590	R	100	426	R	100	100
	CAT	632	H	100	468	H	100	100
	C-terminal	723	Y	100	559	Y	100	100
Camptothecin-resistant mutations	CAP	361	F	100	197	F	100	98.8
	CAP	363	G	100	199	G	100	98.8
	CAP	364	R	100	200	R	100	100
	CAP	418	E	100	254	E	100	97.5
	CAT	503	G	100	339	G	91.8	97.5
	CAT	533	D	100	369	D	95.8	98.8
	Linker	653	A	77.2	489	A	62.8	51.6
	C-terminal	722	N	100	558	N	100	100
	C-terminal	729	T	66.3	565	S	71.6	65.8

The core domain (CAP, Hinge and CAT) is highly conserved due to its fundamental function on DNA binding during catalysis. We also found high conservation in the DNA-binding regions in other topoisomerases (Moreira et al. 2021, 2022), suggesting that these regions cannot accommodate changes due to maintaining the topoisomerase activity through a proper interaction with DNA. The CAT region is slightly more conserved than the CAP region, which agrees with the observation that only the CAT region is conserved in bacterial, viral and eukaryotic topoisomerases (Patel et al. 2010; Perry et al. 2006). The five-residue loop Hinge is conserved across metazoan (84.8% sequence identity), specifically the first two residues (TOP1 positions 428–429) that present the same amino acids in all the analysed species (Fig. 3). In addition, the tyrosine upstream of the Hinge (position 426) was also found conserved, in agreement with a previous work suggesting that this position interacts with the DNA duplex and guides the motion of the CAP domain upon DNA binding to enable the enzyme closing (Takahashi et al. 2022). Within the CAT region, we noticed that near the Linker there are two conserved stretches of around 20 amino acids that flank a poorly conserved region (Fig. 3). In particular, we identified a region with 6 amino acids AKVFRT (TOP1 reference positions 586–591) that is 100% conserved across all the 161 metazoan analysed species. This region includes several active sites. The CAP and CAT regions include sites conferring resistance to camptothecin and all of them are 100% conserved. The only variable sites conferring resistance to camptothecin were observed in the Linker (site 653, sequence identity of 77.2%) and C-terminal (site 729, sequence identity of 66.3%) regions.

We found that the Linker region is more variable than the surrounding core and C-terminal domains (Fig. 3, Table 3). The Linker consists of two long alpha helices connected by a short turn, forming an antiparallel coiled-coil configuration that protrudes away from the remainder of the enzyme (Stewart et al. 1998). We found that its conservation decreases with the increasing distance to the flanking domains and to the catalytic region of the enzyme (Fig. 3). The short turn at the end of the Linker (TOP1 positions 675–678) is extremely variable across species (21.4% of sequence identity), including some variation in length, suggesting that it can vary without affecting the protein function. The increase in conservation of the Linker in regions closer to the core of the enzyme indicates that amino acid replacements are less tolerated if they occur close to the catalytic region, possibly due to affecting the protein activity or the Linker connections to the DNA strand.

The C-terminal domain folds into a globular structure (Figs. 1C, 3) that includes the active-site nucleophile Tyr723 (Redinbo et al. 1998). This region also includes 8 residues near the Linker (718–722) with significant structural similarity with the bacteriophage family of DNA integrases (Redinbo et al. 1998). Our results confirmed previous observations about the high conservation of the C-terminal domain (Champoux 2001). In particular, we found that the 14 amino acids closer to the Linker (human TOP1 positions 715–728) are almost 100% conserved in all the analysed animal species (Fig. 3).

## Conclusions

Type IB topoisomerases are widespread in the animal kingdom. Indeed, vertebrates present specialized topoisomerases to operate with the nuclear and mitochondrial genomes. However, little is known about its evolution and its genetic similarities among species. Here we analysed the molecular evolution of topoisomerases among a variety of animal species. Our phylogenetic investigation placed the event that originated the specialized TOP1 and TOP1MT proteins in the early evolution of vertebrates, possibly associated with whole-genome duplications. After the duplication event, the long-term evolution of both paralogues was primarily driven by strong purifying selection probably to maintain the protein function. However, we found that TOP1MT evolved much faster than TOP1 and other topoisomerases, perhaps related with its specific role within the mitochondria. The fast evolution of TOP1MT was also evident in the missense mutations detected in the Neanderthals, displaying a rare case of protein differences among hominids. Finally, comparison of topoisomerases among species showed that the relevant protein sites for catalytic activities are mainly conserved across animals, again probably caused by their relevant biological roles.

## Supplementary Information

Below is the link to the electronic supplementary material.

Supplementary file1 (PDF 871 KB)

## Data Availability

Not applicable.
