# Supplementary material for: Evolution of TOP1 and TOP1MT Topoisomerases in Chordata
Source: J Mol Evol. 2023 Jan 18;91(2):192–203. doi: 10.1007/s00239-022-10091-z (PMC10081982; doi:10.1007/s00239-022-10091-z)

# SUPLEMENTARY MATERIAL

## Evolution of *TOP1* and *TOP1MT* topoisomerases in Chordata

Filipa Moreira[1,2], Miguel Arenas[3,4,5], Arnaldo Videira[2,6,7] and Filipe Pereira[8,9*]

[1] Interdisciplinary Centre of Marine and Environmental Research (CIIMAR), University of Porto, Terminal de Cruzeiros do Porto de Leixões, Av. General Norton de Matos s/n 4450-208, Matosinhos, Portugal
[2] ICBAS - Instituto de Ciências Biomédicas de Abel Salazar, Universidade do Porto, Rua Jorge de Viterbo Ferreira 228, 4050-313, Porto, Portugal
[3] Department of Biochemistry, Genetics and Immunology, University of Vigo, 36310 Vigo, Spain
[4] CINBIO, Universidade de Vigo, 36310 Vigo, Spain
[5] Galicia Sur Health Research Institute (IIS Galicia Sur), 36310 Vigo, Spain
[6] IBMC-Instituto de Biologia Molecular e Celular, Universidade do Porto, Porto, Portugal
[7] i3S-Instituto de Investigação e Inovação em Saúde, Universidade do Porto, Porto, Portugal
[8] IDENTIFICA genetic testing, Rua Simão Bolívar 259 3º Dir Tras. 4470-214 Maia, Portugal
[9] Centre for Functional Ecology, Department of Life Sciences, University of Coimbra, Calçada Martim de Freitas, 3000-456 Coimbra, Portugal

Supplementary Fig. S1. TOP1 and TOP1MT protein sequences used in this study.

| **Taxonomy** | ***Species - Accession number*** |
|---|---|
| Annelida; Clitellata | *Helobdella robusta - XP_009017579.1* |
| Annelida; Polychaeta | *Capitella teleta - ELU02442.1* |
| | *Owenia fusiformis - CAC9596629.1* |
| | *Dimorphilus gyrociliatus - CAD5111131.1* |
| Arthropoda; Arachnida | *Stegodyphus dumicola - XP_035223075.1* |
| | *Parasteatoda tepidariorum - XP_015914810.1* |
| | *Araneus ventricosus - GBM09797.1* |
| | *Varroa jacobsoni - XP_022703641.1* |
| | *Varroa destructor - XP_022671948.1* |
| | *Rhipicephalus sanguineus - XP_037529830.1* |
| | *Dermacentor silvarum - XP_037558923.1* |
| | *Ixodes scapularis - XP_029851295.2* |
| Arthropoda; Insecta | *Coptotermes formosanus - GFG35199.1* |
| | *Cephus cinctus - XP_015608843.1* |
| | *Polistes dominula - XP_015173950.1* |
| | *Harpegnathos saltator - XP_011140118.2* |
| | *Pogonomyrmex barbatus - XP_011636012.1* |
| | *Formica exsecta - XP_029668836.1* |
| | *Bombus impatiens - XP_024221338.1* |
| | *Bombus pyrosoma - XP_043594904.1* |
| | *Bombus terrestris - XP_012169008.1* |
| | *Tribolium madens - XP_044267953.1* |
| Chordata; Actinopterygii | *Esox lucius - XP_034151999.1_TOP1* |
| | *Esox lucius - XP_010894948.1_MT* |
| | *Danio rerio - XP_002666448.2_TOP1* |
| | *Danio rerio - XP_002665372.2_MT* |
| | *Astyanax mexicanus - XP_022535346.1_MT* |
| | *Astyanax mexicanus - KAG9269888.1_TOP1* |
| | *Pundamilia nyererei - XP_005734805.1_MT* |
| | *Maylandia zebra - XP_004571958.1_TOP1* |
| | *Maylandia zebra - XP_004568268.1_MT* |
| | *Xiphophorus maculatus - XP_005801712.1_TOP1* |
| | *Xiphophorus maculatus - XP_005795820.2_MT* |
| | *Poecilia formosa - XP_007565717.1_TOP1* |
| | *Poecilia formosa - XP_007553331.1_MT* |
| | *Oryzias latipes - XP_023811830.1_MT* |
| | *Oryzias latipes - XP_023810923.1_TOP1* |
| | *Clupea harengus - XP_031421779.1_TOP1* |
| | *Clupea harengus - XP_012671749.1_MT* |
| | *Lepisosteus oculatus - XP_015209608.1_MT* |
| | *Lepisosteus oculatus - XP_006639584.2_TOP1* |
| | *Pundamilia nyererei - XP_005740471.1_TOP1* |

Supplementary Fig. S1. *cont.*

| | |
|---|---|
| Chordata; Amphibia | *Microcaecilia unicolor* - *XP_030075669.1_MT* |
| | *Microcaecilia unicolor* - *XP_030069429.1_TOP1* |
| | *Rhinatrema bivittatum* - *XP_029468354.1_TOP1* |
| | *Rhinatrema bivittatum* - *XP_029448031.1_MT* |
| | *Xenopus laevis* - *XP_018123795.1_MT* |
| | *Xenopus laevis* - *NP_001084031.1_TOP1* |
| | *Xenopus tropicalis* - *XP_031759708.1_MT* |
| | *Xenopus tropicalis* - *XP_002932918.2_TOP1* |
| | *Rana temporaria* - *XP_040208064.1_MT* |
| | *Rana temporaria* - *XP_040186339.1_TOP1* |
| Chordata; Aves | *Dromaius novaehollandiae* - *XP_025969476.1_MT* |
| | *Dromaius novaehollandiae* - *XP_025948785.1_TOP1* |
| | *Melopsittacus undulatus* - *XP_033922770.1_TOP1* |
| | *Melopsittacus undulatus* - *XP_033918251.1_MT* |
| | *Fulmarus glacialis* - *XP_009585324.1_TOP1* |
| | *Fulmarus glacialis* - *XP_009575693.1_MT* |
| | *Chiroxiphia lanceolata* - *XP_032569132.1_MT* |
| | *Chiroxiphia lanceolata* - *XP_032560816.1_TOP1* |
| | *Ficedula albicollis* - *XP_005057170.1_TOP1* |
| | *Ficedula albicollis* - *XP_005042662.1_MT* |
| | *Meleagris gallopavo* - *XP_010720624.1_TOP1* |
| | *Meleagris gallopavo* - *XP_010707646.1_MT* |
| | *Cygnus olor* - *XP_040431480.1_TOP1* |
| | *Cygnus olor* - *XP_040403374.1_MT* |
| | *Cygnus atratus* - *XP_035422285.1_MT* |
| | *Cygnus atratus* - *XP_035403913.1_TOP1* |
| | *Oxyura jamaicensis* - *XP_035174776.1_MT* |
| | *Columba livia* - *XP_021143570.1_TOP1* |
| | *Columba livia* - *XP_005504308.1_MT* |
| | *Calidris pugnax* - *XP_014821120.1_MT* |
| | *Calidris pugnax* - *XP_014796749.1_TOP1* |
| | *Oxyura jamaicensis* - *XP_035199725.1_TOP1* |
| Chordata; Cephalochordata | *Branchiostoma lanceolatum* - *CAH1270849.1* |
| | *Branchiostoma floridae* - *XP_035678657.1* |
| | *Branchiostoma belcheri* - *XP_019646913.1* |
| Chordata; Chondrichthyes | *Callorhinchus milii* - *XP_042197656.1_TOP1* |
| | *Chiloscyllium plagiosum* - *XP_043566649.1_TOP1* |
| | *Chiloscyllium plagiosum* - *XP_043543828.1_MT* |
| | *Carcharodon carcharias* - *XP_041059975.1_TOP1* |
| | *Carcharodon carcharias* - *XP_041045416.1_MT* |
| Chordata; Coelacanthimorpha | *Latimeria chalumnae* - *XP_014352319.1* |
| | *Latimeria chalumnae* - *XP_005990473.1* |
| Chordata; Cyclostomata | *Petromyzon marinus* - *XP_032816127.1_MT* |
| | *Petromyzon marinus* - *XP_032813624.1_TOP1* |

Supplementary Fig. S1. *cont.*

| | |
|---|---|
| Chordata; Mammalia | *Carlito syrichta* - XP_008058764.1_TOP1 |
| | *Carlito syrichta* - XP_008049557.1_MT |
| | *Nomascus leucogenys* - XP_003280801.2_MT |
| | *Nomascus leucogenys* - XP_003253628.1_TOP1 |
| | *Pongo abelii* - XP_024106654.1_MT |
| | *Pongo abelii* - XP_024094656.1_TOP1 |
| | *Pan paniscus* - XP_008971078.2_MT |
| | *Homo sapiens* - NP_443195.1_MT |
| | *Homo sapiens* - NP_003277.1_TOP1 |
| | *Gorilla gorilla gorilla* - XP_004047664.2_MT |
| | *Rhinopithecus roxellana* - XP_030777108.1_MT |
| | *Rhinopithecus roxellana* - XP_010374253.1_TOP1 |
| | *Papio anubis* - XP_031525665.1_MT |
| | *Papio anubis* - XP_021776748.1_TOP1 |
| | *Macaca mulatta* - XP_015001609.2_MT |
| | *Macaca mulatta* - NP_001253441.1_TOP1 |
| | *Cercocebus atys* - XP_011921036.1_TOP1 |
| | *Cercocebus atys* - XP_011905464.1_MT |
| | *Pan paniscus* - XP_003825942.1_TOP1 |
| | *Gorilla gorilla* - XP_004062202.1_TOP1 |
| Chordata; Reptilia | *Pelodiscus sinensis* - XP_014433332.2_MT |
| | *Pelodiscus sinensis* - XP_006121946.1_TOP1 |
| | *Gopherus evgoodei* - XP_030409772.1_MT |
| | *Gopherus evgoodei* - XP_030389046.1_TOP1 |
| | *Chelonoidis abingdonii* - XP_032623226.1_TOP1 |
| | *Chelonoidis abingdonii* - XP_032619134.1_MT |
| | *Mauremys mutica* - XP_044859482.1_MT |
| | *Mauremys mutica* - XP_044841689.1_TOP1 |
| | *Trachemys scripta elegans* - XP_034643158.1_TOP1 |
| | *Trachemys scripta elegans* - XP_034615486.1_MT |
| | *Chrysemys picta bellii* - XP_023958405.2_MT |
| | *Chrysemys picta bellii* - XP_008166335.2_TOP1 |
| | *Dermochelys coriacea* - XP_038249347.1_MT |
| | *Dermochelys coriacea* - XP_038225734.1_TOP1 |
| | *Chelonia mydas* - XP_027687421.1_MT |
| | *Chelonia mydas* - XP_007057244.1_TOP1 |
| Chordata; Tunicata | *Styela clava* - XP_039265207.1 |
| | *Ciona intestinalis* - XP_026693862.1 |
| | *Phallusia mammillata* - CAB3267168.1 |
| | *Oikopleura dioica* - CAG5098597.1 |
| Cnidaria; Anthozoa | *Pocillopora damicornis* - XP_027046231.1 |
| | *Acropora millepora* - XP_029192757.2 |
| | *Nematostella vectensis* - XP_032231206.1 |
| | *Actinia tenebrosa* - XP_031560302.1 |

Supplementary Fig. S1. *cont.*

| | |
|---|---|
| Echinodermata; Asteroidea | *Patiria miniata* - *XP_038077725.1* |
| | *Acanthaster planci* - *XP_022094493.1* |
| | *Asterias rubens* - *XP_033625993.1* |
| Echinodermata; Crinoidea | *Anneissia japonica* - *XP_033125260.1* |
| Echinodermata; Echinoidea | *Lytechinus variegatus* - *XP_041454051.1* |
| | *Strongylocentrotus purpuratus* - *XP_030831651.1* |
| Echinodermata; Holothuroidea | *Apostichopus japonicus* - *PIK62638.1* |
| Hemichordata; Enteropneusta | *Saccoglossus kowalevskii* - *XP_006814310.1* |
| Mollusca; Bivalvia | *Pecten maximus* - *XP_033754799.1* |
| | *Mytilus edulis* - *CAG2232811.1* |
| | *Dreissena polymorpha* - *KAH3774199.1* |
| Mollusca; Cephalopoda | *Octopus bimaculoides* - *XP_014781973.1* |
| Mollusca; Gastropoda | *Haliotis rubra* - *XP_046570009.1* |
| | *Aplysia californica* - *XP_005091292.1* |
| | *Elysia chlorotica* - *RUS80862.1* |
| | *Biomphalaria glabrata* - *XP_013086265.1* |
| | *Batillaria attramentaria* - *KAG5707923.1* |
| | *Pomacea canaliculata* - *XP_025076151.1* |
| Nematoda; Enoplea | *Trichinella pseudospiralis* - *KRY68761.1* |
| | *Trichinella papuae* - *KRZ70158.1* |
| | *Trichinella britovi* - *KRY50853.1* |
| Platyhelminthes; Cestoda | *Echinococcus granulosus* - *KAH9281024.1* |
| | *Echinococcus multilocularis* - *CDS39478.1* |
| | *Taenia asiatica* - *VDK20333.1* |
| Platyhelminthes; Trematoda | *Paragonimus heterotremus* - *KAF5397961.1* |
| | *Clonorchis sinensis* - *KAG5443823.1* |
| | *Fasciolopsis buski* - *KAA0200075.1* |
| | *Fasciola gigantica* - *TPP66131.1* |
| Porifera; Demospongiae | *Amphimedon queenslandica* - *XP_011404857.2* |
| Tardigrada; Eutardigrada | *Ramazzottius varieornatus* - *GAU96790.1* |
| | *Hypsibius dujardini* - *OQV12457.1* |

Supplementary Fig. S2. Alignment of Neanderthal sequence reads against the human *TOP1MT* reference sequence. The two missense mutations identified in the Neanderthal lineage and indicated. The top alignment was done by us using the sequences downloaded from the UCSC Genome Browser. The bottom image is a snapshot of the assembly available at The Neandertal Genome Project (http://neandertal.ensemblgenomes.org).

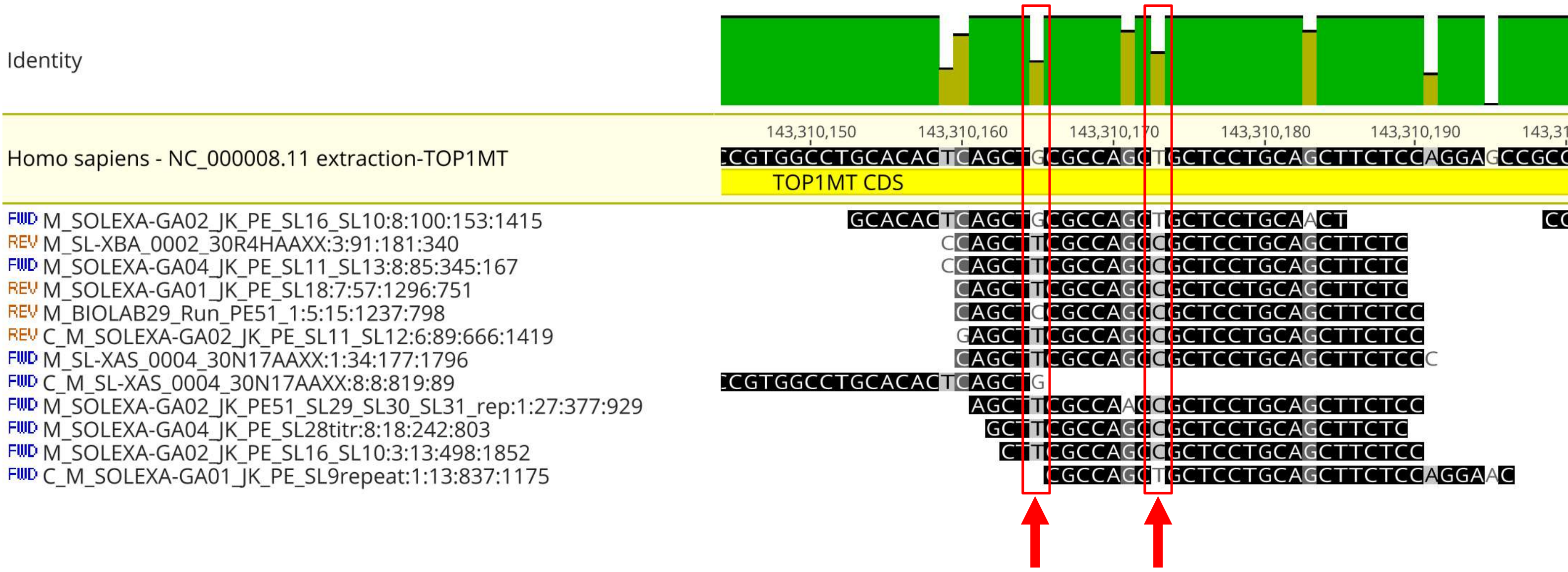


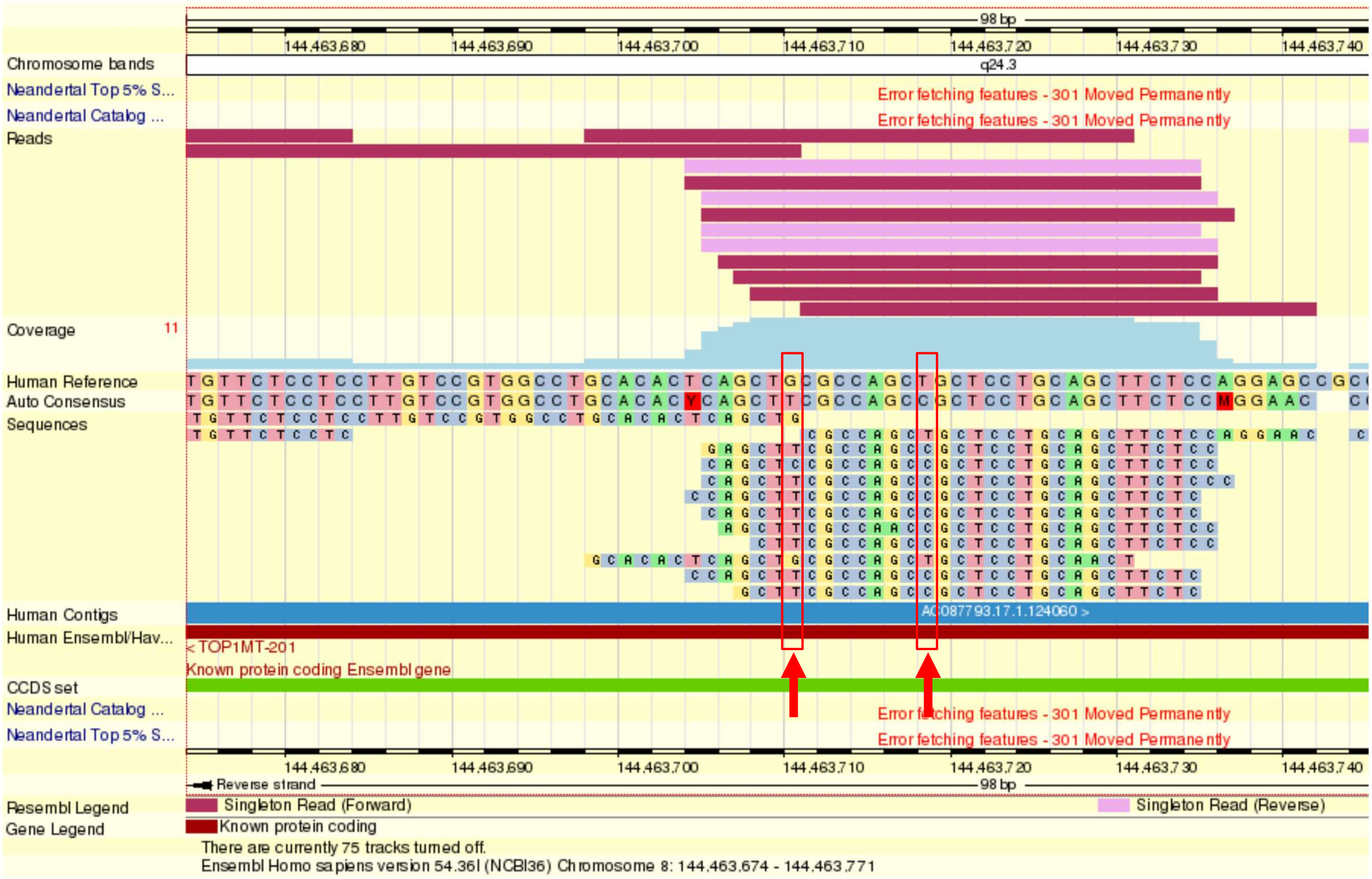

Supplement: Supplementary file 1 — Supplementary file1 (PDF 871 KB) [file 239_2022_10091_MOESM1_ESM.pdf]
